# Large-Scale Survey for Tickborne Bacteria, Khammouan Province, Laos

**DOI:** 10.3201/eid2209.151969

**Published:** 2016-09

**Authors:** Andrew J. Taylor, Khamsing Vongphayloth, Malavanh Vongsouvath, Marc Grandadam, Paul T. Brey, Paul N. Newton, Ian W. Sutherland, Sabine Dittrich

**Affiliations:** Mahosot Hospital, Vientiane, Laos (A.J. Taylor, M. Vongsouvath, P.N. Newton, S. Dittrich);; University of Oxford, Oxford, UK (A.J. Taylor, P.N. Newton, S. Dittrich);; Institut Pasteur du Laos, Vientiane (K. Vongphayloth, M. Grandadam, P.T. Brey, I.W. Sutherland);; US Naval Medical Research Center–Asia, Sembawang, Singapore (I.W. Sutherland)

**Keywords:** ticks, tickborne bacteria, bacteria, vector-borne infections, Anaplasma, Ehrlichia, Coxiella, Rickettsia, Borrelia, Amblyomma, Haemaphysalis, Dermacentor, survey, Southeast Asia, Laos

## Abstract

We screened 768 tick pools containing 6,962 ticks from Khammouan Province, Laos, by using quantitative real-time PCR and identified *Rickettsia* spp., *Ehrlichia* spp., and *Borrelia* spp. Sequencing of *Rickettsia* spp.–positive and *Borrelia* spp.–positive pools provided evidence for distinct genotypes. Our results identified bacteria with human disease potential in ticks in Laos.

*Rickettsia*, *Borrelia*, *Ehrlichia, Anaplasma*, and *Coxiella* spp. are tick-associated bacteria and well-described human pathogens. All of these bacteria, except *Coxiella* spp., are primarily transmitted through tick bites and cause febrile disease with a wide spectrum of severity. Tickborne bacterial pathogens are believed to be an underrecognized cause of acute febrile illness in Southeast Asia (*1*).

In Laos, spotted fever group *Rickettsia* have been shown to cause undifferentiated fever in 2% of febrile hospitalized adult patients (*2*). However, data on bacteria in ticks in Laos are sparse. To date, 1 *Rickettsia* sp. has been identified in a *Boophilus* sp. tick from Luang Namtha Province; this species showed 99.8% similarity with the *Rickettsia* sp. FUJ98 *ompA* gene (*3*). No other tickborne bacteria have been reported from Laos. Therefore, we investigated *Rickettsia*, *Borrelia*, *Ehrlichia*, *Anaplasma*, and *Coxiella* spp. in ticks from Khammouan Province, Laos.

## The Study

We collected ticks in Nakai District, Khammouan Province, during the dry seasons (December–April) during 2012–2014, as previously described (*4*) (Technical Appendix Figures 1, 2). A total of 6,692 ticks were pooled (n = 768 pools, 1–10 ticks/pool) according to genus, sex, developmental stage, collection period, and site. One *Amblyomma testudinarium* nymph that contained a blood meal was processed separately.

We extracted DNA by using the NucleoSpin 8 Virus Extraction Kit (Macherey-Nagel, Düren, Germany). Pools were screened by using single quantitative real-time PCRs specific for *Rickettsia* spp. (17-kDa gene), *Borrelia* spp. (23S rRNA gene), *Anaplasma* spp. (major surface protein 2 gene), *Ehrlichia* spp. (16S rRNA gene), and *Coxiella* spp. (IS1111) (*5*–*8*) (Technical Appendix Table 1). Five microliters of diluted (1:10) template containing 1× Platinum Supermix-UDG (Invitrogen, Carlsbad, CA, USA) and bovine serum albumin (40 mg/mL) were used for each assay. Positive and nontemplate controls were included in each run. Screening by PCR was performed once per sample. In concordance with published guidelines, results were considered positive if they had a cycle quantitation (C_q_) value <40 and likely positive if they had a C_q_ value 40–45 (*9*).

Sequencing was attempted for pools with C_q_ values <40 (online Technical Appendix Table 2) and performed by Macrogen (Seoul, South Korea). Consensus sequences were analyzed by using CLC Main Workbench 7 (http://www.clcbio.com/products/clc-main-workbench/) and BLAST (http://blast.ncbi.nlm.nih.gov/Blast.cgi) and submitted to GenBank. Phylogenetic trees were constructed by using the Kimura-2 parameter model and the neighbor-joining method. Bootstrap values were determined by using 1,000 replications.

**Table 2:** Sequence data for *Rickettsia* species isolated from ticks, Khammouan Province, Laos*

Tick pool	Tick species and stage	*Rickettsia* spp. gene, GenBank accession no., and % similarity (no. matching nucleotides/total)				
		17-kDa	*gltA*	*sca4*	*ompA*	*ompB*
110	*Amblyomma testudinarium *nymph	Unclear sequence	NS	Unclear sequence	KT753264, 100.0 (529/529) with *Rickettsia *sp. TwKM01 EF219467	NS
177, 180, 216, 220	*A. testudinarium *nymph	KR733070, 100.0 (355/355) with *R. tamurae *AB114825	KT753265, 99.8 (1,096/1,098) with *R. tamurae* AB812551	KT753266, 99.7 (607/609) with *R. tamurae* DQ113911	NS	NS
315	*A. testudinarium* nymph	KT753267, 98.8 (407/412) with *R. raoultii *JX885457	KT753268, 99.9 (1,036/1,037) with *Ricksettia *kagoshima6 JQ697956	KT753269, 96.8 (795/821) with *Rickettsia *sp. AUS 118, KF666473	Could not be amplified	KT753270, 95.0 (1,073/1,129) with *R. massiliae* CP003319
239	*A. testudinarium* nymph	KT753271, 99.7 (360/361) with *Rickettsia *sp. ATT AF483196	KT753272, 99.7 (1,048/1,051) with *R. tamurae* (AB812551)/KT753273; 99.2 (367/370) with *Rickettsia *sp. hhmj7 KC566999	KT753274, 97.1 (759/782) with *Rickettsia *sp. AUS 118 KF666473	KT753275, 87.2 (530/602) with *R. raoultii* JQ792137	KT753276, 97.5 (1,052/1,079) with *R. massiliae* CP003319
76, 337, 450, 453	*Haemaphysalis* G1 nymphs (3), *A. testudinarium* nymph (1)	KT753277, 98.4 (417/423) with *R. raoultii* JX885457	KT753278, 99.9 (1,037/1,038) with *Ricksettia *sp. kagoshima6 JQ697956	KT753279, 98.4 (794/807) with *R. japonica *AF155055	Could not be amplified	KT753280, 96.0 (410/427) with *R. raoultii* EU036984
81, 372	*Haemaphysalis* G1 nymphs, *A. testudinarium* nymph (17 kDa only)	KT753283, 99.0 (408/412) with *R. raoultii* JX885457	KT753284, 99.5 (1,090/1,096) with *R. sibirica* U59734	KT753285, 98.5 (838/851) with *R. japonica* AF 155055	Could not be amplified	KT753286, 97.7 (1,118/1,144) with *R. massiliae* CP003319
120	*Haemaphysalis* G1 nymph	KT753287, 96.1 (391/407) with *R. helvetica* GU827073	KT753288, 97.1 (370/381) with *Candidatus *Rickettsia rara DQ365805	Could not be amplified (x2)	Could not be amplified (x2)	KT753289, 86.4 (362/419), *R. aeschlimannii* AF123705
407	*Haemaphysalis hysticis* adult	KR733074, 100.0 (413/413), *R. japonica* AP011533	KT753281, 100.0 (1,063/1,063), *R. japonica* AP011533	NS	NS	KT753282, 100.0 (1,191/1,191) with *R. japonica *AP011533
447	*Haemaphysalis* G1 nymph	KT753291, 98.6 (407/413) with *R. massiliae* CP000683	KT753290, 99.6 (961/965) with *R. raoultii* JX885455	KT753292, 97.5 (809/830) with *Rickettsia *sp. AUS118 KF66473	KT753293, 97.5 (591/606) with *Rickettsia *sp. JL-02 AY093696	KT753294, 98.4 (1,137/1,156), with *R. massiliae* CP003319

*New sequences were compared with reference sequences. NS, not sequenced.

A total of 768 tick pools containing 6,692 ticks were screened. Pools contained 3 genera of ticks: 59.9% (460/768) *Haemaphysalis* spp., 36.3% (279/768) *A. testudinarium*, and 3.8% (29/768) *Dermacentor auratus*. Of the pools, 3% (23/768) contained adults, 36.5% (280/768) contained larvae, and 60.5% (465/768) contained nymphs (Table 1).

**Table 1:** Tick pools tested for bacteria after screening by quantitative PCR, Khammouan Province, Laos*

Bacteria and tick species	No. positive pools/no. tested (%)				
	Total	Larvae	Nymphs	Adult males	Adult females
*Rickettsia* spp.					
All	44/768 (5.7)	6/280 (2.1)	37/465 (8.2)	0/12 (0)	1/11 (9.1)
* Amblyomma testudinarium*	27/279 (10.0)	0/61 (0)	27/217 (12.9)	0/1 (0)	0/1 (0)
* Haemaphysalis G1*	5/398 (3.8)	6/194 (3.1)	9/200 (4.5)	0/3 (0)	0/1 (0)
* H. hystricis*	1/6 (16.7)	NS	NS	0/3 (0)	1/3 (33.3)
* Dermacentor auratus*	1/29 (3.4)	0/0 (0)	1/26 (3.8)	0/2 (0)	0/1 (0)
*Ehrlichia* spp.					
All	12/768 (1.6)	4/280 (1.4)	6/465 (1.3)	1/12 (8.3)	1/11 (9.1)
* A. testudinarium*	2/279 (0.7)	0/61 (0)	2/217 (0.9)	0/1 (0)	0/1 (0)
* Haemaphysalis G1*	8/398 (2.0)	4/194 (2.1)	4/200 (2.0)	0/3 (0)	0/1 (0)
* H. aborensis*	2/6 (33.3)	NS	NS	1/3 (33.3)	1/3 (33.3)
*Borrelia* spp.					
All	12/768 (1.6)	2/280 (0.7)	8/465 (1.7)	2/12 (16.7)	NS
* A. testudinarium*	2/279 (0.7)	1/61 (1.6)	1/217 (0.5)	0/1 (0)	0/1 (0)
* Haemaphysalis G1*	6/398 (1.5)	1/194 (0.5)	5/200 (2.5)	0/3 (0)	0/1 (0)
* Haemaphyalis G1.2*	1/13 (7.7)	NS	1/13 (7.7)	NS	NS
* H. aborensis*	2/6 (33.3)	NS	NS	2/3 (66.7)	0/3 (0)
* D. auratus*	1/29 (3.4)	0/0 (0)	1/26 (3.8)	0/2 (0)	0/1 (0)
*Coxiella *spp.					
All	5/511 (1.0)†	4/187 (2.1)†	1/310 (0.3)	0/8 (0)	0/6 (0)
* Haemaphysalis G1*	5/279 (1.8)†	4/162 (2.5)†	1/117 (0.9)	NS	NS
*Anaplasma* spp.					
All	2/768 (0.3)†	0/280 (0)†	0/465 (0)†	0/12 (0)	0/11 (0)
* A. testudinarium*	1/279 (0.4)†	0/61 (0)	1/217 (0.5)†	0/1 (0)	0/1 (0)
* Haemaphysalis G1*	1/398 (0.3)†	1/194 (0.5)†	0/200 (0)	0/3 (0)	0/1 (0)


*NS, no samples were available for screening. †Includes samples with cycle quantitation values <40 and 40–45.

**Figure 1:**
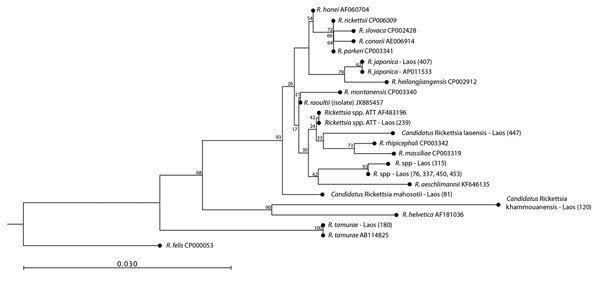
Phylogenetic analysis of *Rickettsia* spp. in ticks, Khammouan Province, Laos. The tree was constructed by using partial nucleotide sequences (350 bp) of the 17-kDa gene, the Kimura 2-parameter model, and the neighbor-joining method. Analyses were supported by bootstrap analysis with 1,000 replications. Numbers along branches are bootstrap values. GenBank accession numbers are shown for reference sequences. Sample numbers for each tick are shown in parentheses. Scale bar indicates nucleotide substitutions per site.

*Rickettsia* spp. were identified in 5.7% (44/768) of pools, and an additional 2.3% (18/768) of pools were likely positive for *Rickettsia* spp. Sequences consistent with 5 described *Rickettsia* species or genotypes were identified: *R. tamurae, R. japonica, Rickettsia* sp. ATT, *Rickettsia* sp. Kagoshima6, and *Rickettsia* sp. TwKM01 (Table 2; Figure 1).

Three novel genotypes (Table 2) were identified that might be new species. *Candidatus* Rickettsia laoensis (pool 447) was identified in 1 *Haemaphysalis* sp. pool. Phylogenetic analysis of 2845–2920-bp concatenated sequences of *gltA, sca4*, and *ompB* genes suggested that this bacteria belonged to the *R. massiliae* group of rickettsiae (Technical Appendix Figure 3). *Candidatus* Rickettsia mahosotii (pools 81 and 372) was identified in *Haemaphysalis* spp. and *A. testudinarium* pools. Phylogenetic analysis of *gltA, sca4*, and *ompB* genes suggested that this bacteria belonged to the *R. rickettsii* group (Technical Appendix Figure 3). *Candidatus* Rickettsia khammouanensis was identified in 1 *Haemaphysalis* sp. nymph pool (pool 120). Phylogenetic analysis of *gltA,* 17-kDa, and *ompB* genes suggested a relationship with the *R. helvetica* group (Technical Appendix Figure 4).

In addition, 15 *A. testudinarium* pools showed dual peaks for 17-kDa gene sequences, which suggested the presence of *R. tamurae* and *Rickettsia* sp. ATT. Sequencing of *sca4, ompA*, and *ompB* genes from 1 of these pools (pool 239) identified unique sequences (Table 2; Technical Appendix Figure 4).

**Figure 2:**
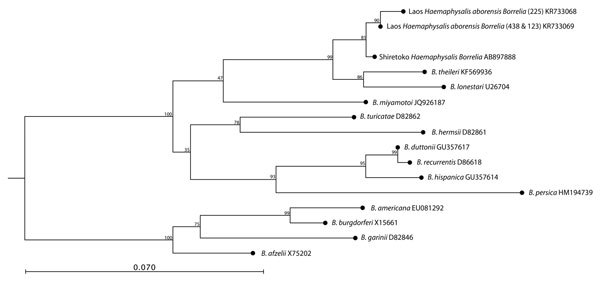
Phylogenetic analysis of *Borrelia* spp. in ticks, Khammouan Province, Laos. The tree was constructed by using partial nucleotide sequences (299–323 bp) of the *flaB* gene, the Kimura 2-parameter model, and the neighbor-joining method. Analyses were supported by bootstrap analysis with 1,000 replications. Numbers along branches are bootstrap values. GenBank accession numbers are shown for reference sequences. Sample numbers for each tick are shown in parentheses. Scale bar indicates nucleotide substitutions per site.

*Borrelia* spp. were identified in 1.6% (12/768) of pools (Table 1). Two unique sequences obtained from *Haemaphysalis* spp. pools showed 99.3% (298/300) (GenBank accession no. KR733069) and 98.7% (296/300) (accession no. KR733068) identity with Shiretoko *Haemaphysalis Borrelia* sp. (AB897888). Phylogenetic analysis confirmed that both bacteria were closely related to Shiretoko *Haemaphysalis Borrelia* sp. (accession no. B897888) and belong to the relapsing fever group of *Borrelia* (Figure 2).

Twelve (1.6%) of 768 pools were positive for *Ehrlichia* spp. (Table 1); an additional 6 pools (0.8%) were likely positive. One short sequence from a *Haemaphysalis* sp. nymph pool (pool 357) was obtained, and this sequence showed 100% identity (116/116 bases) with the genus *Ehrlichia*.

No pools were positive for *Anaplasma* spp., but 2 were likely positive (Table 1). Although not all pools were tested for *Coxiella* spp. (n = 511), 1 pool (0.2%) was positive, and 4 pools were likely positive for *C. burnetti*. No confirmatory sequences were obtained from these pools. The 1 tick that contained a blood meal (*A. testudinarium* nymph) showed negative results by screening PCRs.

## Conclusions

This study provides evidence that *Rickettsia* spp., *Borrelia* spp., and *Ehrlichia* spp. are present in ticks in Laos. Several *Rickettsia* spp. identified in this study are human pathogens. Infections with *R. tamurae* (*2*) and *R. japonica* are well described in Southeast Asia (*10*). However, the pathogenicity of *Rickettsia* sp. TwkM01 (*11*), *Rickettsia* sp. ATT (*12*), *Rickettsia* sp. kagoshima6 genotypes (*13*) and potential novel *Candidatus* Rickettsia laoensis, *Candidatus* Rickettsia mahosotii, and *Candidatus* Rickettsia khammouanensis is unknown. *Candidatus* Rickettsia khammouanensis is phylogenetically related to *R. helvetica*, for which there is serologic evidence for its role as a human pathogen in Laos (*2*). Unique *ompA*, *ompB*, and *sca4* sequences identified in this study (Table 2) might indicate the presence of *Rickettsia* sp. ATT (*12*), which was previously believed to be identical to *R. tamurae* (*14*), and suggests that it might be a distinct species. Further studies, including whole-genome sequencing, are required to identify and confirm these novel genotypes and understand their role in human disease.

*Borrelia* spp. sequences identified in *Haemaphysalis* spp. pools were shown to have high concordance with the Shiretoko *Haemaphysalis Borrelia* isolated from *Haemaphysalis* spp. ticks and deer in Japan (*15*). The species belongs to the relapsing fever group of *Borrelia* and is related to *B. lonestari*.

Sequence data for *Ehrlichia* spp. indicated the presence of these bacteria but were not sufficient to identify them to the species level. The C_q_ values were high (40–45) for *Anaplasma* spp., but no sequence data were obtained. *Coxiella* spp. were screened by using primers for IS1111, which are not specific for *C. burnetii*, and no confirmatory sequence data were obtained. Because of limited reagents, screening of all 768 pools for *Coxiella* spp. was not completed. Further work is required to investigate the presence of these bacteria in Laos.

Our study had several limitations. First, pooling of ticks precludes an accurate assessment of prevalence of bacterial pathogens. Second, sequences obtained from some *A. testudinarium* pools had dual peaks, suggestive of multiple infections, and could therefore not be interpreted. Third, ticks were collected only from 1 area in Laos (Khammouan Province); thus, extrapolating findings to the entire country must be done cautiously.

Our results highlight the frequency of tickborne bacterial infections in Laos. These findings emphasize the need for further research of tick-associated bacteria and their role in human disease.

Technical Appendix Additional information on large-scale survey for tickborne bacteria, Khammouan Province, Laos.
