# Correction: Vol. 22, No. 9

**DOI:** 10.3201/eid2211.C12211

**Published:** 2016-11

**Keywords:** errata, erratum, correction

Some descriptions of tickborne transmission of bacteria were unclear in Large-Scale Survey for Tickborne Bacteria, Khammouan Province, Laos (A.J. Taylor al.). The article has been corrected online (http://wwwnc.cdc.gov/eid/article/22/9/15-1969_article).

