# Supplementary material for: Large-Scale Survey for Tickborne Bacteria, Khammouan Province, Laos
Source: Emerg Infect Dis. 2016 Sep;22(9):1635–9. doi: 10.3201/eid2209.151969 (PMC4994350; doi:10.3201/eid2209.151969)
Supplement: Technical Appendix — Additional information on large-scale survey for tickborne bacteria, Khammouan Province, Laos. [file 15-1969-Techapp-s1.pdf]

Article DOI: http://dx.doi.org/10.3201/eid2209/151969

Large-Scale Survey for Tickborne Bacteria, Khammouan Province, Laos

Technical Appendix

Technical Appendix Table 1. Primers and probes used for screening quantitative PCR in large-scale survey for tickborne bacteria, Khammouan Province, Laos*

	Primers and probes used to screen samples			
Bacteria	Gene	Primer or probe	Sequence, 5′→3′	Reference†
Rickettsia spp.	17 kDa	R17K128F2	F-GGGCGGTATGAAYAAACAAG	(*5*)
Spotted fever group	17 kDa	R17K238R	R-CCTACACCTACTCCVACAAG	(*5*)
Spotted fever group	17 kDa	R17K202TAQP	P-CCGAATTGAGAACCAAGTAATGC	(*5*)
Borrelia spp.	23S rRNA	Bb23Sf	F-CGAGTCTTAAAAGGGCGATTTAGT	(*6*)
Borrelia spp.	23S rRNA	Bb23Sr	R-TATTTATGGCCAGGCTGAAGC	(*6*)
Borrelia spp.	23S rRNA	Bb23Sp	P-AGATGTGGTAGACCCGAAGCCGAGTG	(*6*)
Coxiella spp.	IS1111	IS1111f	F-CAAGAAACGTATCGCTGTGGC	(*7*)
Coxiella spp.	IS1111	IS1111R	R-CACAGAGCCACCGTATGAATC	(*7*)
Coxiella spp.	IS1111	IS1111 probe	P-CCGAGTTCGAAACAATGAGGGCTG	(*7*)
Ehrlichia spp.	16S rRNA	EHR16S-17	F-GCGGCAAGCCTAACACAT	(*8*)
Ehrlichia spp.	16S rRNA	EHR16S-97	R-CCCGTCTGCCACTAACAATTATT	(*8*)
Ehrlichia spp.	16S rRNA	EHR16S-38	P-AGTCGAACGGACAATTGCTTATAACCTTTTGGT	(*8*)
Anaplasma spp.	msp2	ApMSP2f	F-ATGGAAGGTAGTGTTGGTTATGGTATT	(*6*)
Anaplasma spp.	msp2	APMSP2r	R-TTGGTCTTGAAGCGCTCGTA	(*6*)
Anaplasma spp.	msp2	ApMSP2p	P-TGGTGCCAGGGTTGAGCTTGAGATTG	(*6*)

*F, forward; IS, insertion sequence; msp2, major surface protein 2; P, probe; R, reverse.
†References are in the text of the article.

Technical Appendix Table 2. Primers used for sequencing in large-scale survey for tickborne bacteria, Khammouan Province, Laos*

		Primers used to sequence bacteria-positive samples			
Bacteria	Gene	Primer	Sequence, 5′→3′	Size, bp	Reference
Rickettsia spp.	17 kDa	R17kM61F†	F-ACTTTACAAAATTCTAAAAACCATATACT	524	(*1*)
Spotted fever group	17 kDa	R17K31F‡	F-CATTGTCCGTCAGGTTGGCG	524	(*1*)
Spotted fever group	17 kDa	Rr2608Rnew†‡	R-CATTGTCCGTCAGGTTGGCG	434	(*1*)
Spotted fever group	*gltA*	Cs1dF†‡	F-ATGACTAATGGCAATAATAA	1,237	(*2*)
Spotted fever group	*gltA*	RpE CS877p‡	F-CATAACCAGTGTAAAGCTG	1,237	(*2*)
Spotted fever group	*gltA*	CS1273R†	R-GGGGGCCTGCTCACGGCGG	382	(*2*)
Spotted fever group	*gltA*	RpE CS1258n‡	R-ATTGCAAAAAGTACAGTGAACA	382	(*2*)
Spotted fever group	*sca4*	RrD749F†‡	F-TGGTAGCATTAAAAGCTGATGG	1,078	(*3*)
Spotted fever group	*sca4*	RrD928F‡	F-ATTTATACACTTGCGGTAACAC	1,078	(*3*)
Spotted fever group	*sca4*	RrD1826R†‡	R-TCTAAATKCTGCTGMATCAAT	899	(*3*)
Spotted fever group	*ompA*	RompAM50F†	R-TTGCGTTATAACACTTTTTAAGTGA	692	(*4*)
Spotted fever group	*ompA*	190–70F‡	F-ATGGCGAATATTTCTCCAAAA	692	(*4*)
Spotted fever group	*ompA*	RompA642R†	R-ATTACCTATTGTTCCGTTAATGGCA	632	(*4*)
Spotted fever group	*ompA*	190–701R‡	R-GTTCCGTTAATGGCAGCATCT	632	(*4*)
Spotted fever group	*ompB*	RompB11F†‡	F-ACCATAGTAGCMAGTTTTGCAG	1,902	(*5*)
Spotted fever group	*ompB*	120–607F‡	F-AATATCGCTGACGGTCAAGGT	1,902	(*5*)
Spotted fever group	*ompB*	RompB1902R†‡	R-CCGTCATTTCCAATAACTAACTC	1,265	(*5*)
Spotted fever group	*ompB*	RAK1452R‡	R-SGTTAACTTKACCGYTTATAACTGT	1,452	(*5*)
Borrelia spp.	*fla B*	280F†	F-GCAGTTCARTCAGGTAACGG	1,452	(*6*)
Borrelia spp.	*fla B*	754R†	R-TAGCAAGTGATGTATTRGCATCAAC	475	(*6*)
Borrelia spp.	*fla B*	301F‡	F-ACATATTCAGATGCAGACAGAGG	475	(*6*)
Borrelia spp.	*fla B*	737R‡	R-GCATCAACTGTRGTTGTAACATTAACAGG	437	(*6*)
Ehrlichia spp.	16 S rRNA	Ehr16SF	F-GTACCYACAGAAGAAGTCC	437	(*7*)
Ehrlichia spp.	16 S rRNA	Ehr16SR	R-GCTGTAAACGATGAGTGCTAA	345	(*7*)

*F, forward; R, reverse.
†First reaction.
‡Second reaction.

References

1. Maina A. Sero-epidemiology and molecular characterization of Rickettsiae infecting humans, selected animals and arthropod vectors in Asembo, western Kenya, 2007–2010 [Doctoral dissertation]. Nairobi (Kenya): Jomo Kenyatta University of Agriculture and Technology; 2012.

2. Roux V, Rydkina E, Eremeeva M, Raoult D. Citrate synthase gene comparison, a new tool for phylogenetic analysis, and its application for the rickettsiae. Int J Syst Bacteriol. 1997;47:252–61. PubMed http://dx.doi.org/10.1099/00207713-47-2-252

3. Jiang J, Sangkasuwan V, Lerdthusnee K, Sukwit S, Chuenchitra T, Rozmajzl P, et al. Human infection with *Rickettsia honei* Thailand. Emerg Infect Dis. 2005;11:1473–5. PubMed http://dx.doi.org/10.3201/eid1109.050011

4. Fournier PE, Roux V, Raoult D. Phylogenetic analysis of spotted fever group rickettsiae by study of the outer surface protein rOmpA. Int J Syst Bacteriol. 1998;48:839–49. PubMed http://dx.doi.org/10.1099/00207713-48-3-839

5. Roux V, Raoult D. Phylogenetic analysis of members of the genus *Rickettsia* using the gene encoding the outer-membrane protein rOmpB (*ompB*). Int J Syst Evol Microbiol. 2000;50:1449–55. PubMed http://dx.doi.org/10.1099/00207713-50-4-1449

6. Clark KL, Leydet B, Hartman S. Lyme borreliosis in human patients in Florida and Georgia, USA. Int J Med Sci. 2013;10:915–31. PubMed http://dx.doi.org/10.7150/ijms.6273

7. Dittrich S, Phuklia W, Turner GD, Rattanavong S, Chansamouth V, Dumler SJ, et al. *Neorickettsia sennetsu* as a neglected cause of fever in South-East Asia. PLoS Negl Trop Dis. 2015;9:e0003908. PubMed http://dx.doi.org/10.1371/journal.pntd.0003908

8. Phongmany S, Rolain JM, Phetsouvanh R, Blacksell SD, Soukkhaseum V, Rasachack B, et al. Rickettsial infections and fever, Vientiane, Laos. Emerg Infect Dis. 2006;12:256–62. PubMed http://dx.doi.org/10.3201/eid1202.050900

9. Kernif T, Socolovschi C, Wells K, Lakim MB, Inthalad S, Slesak G, et al. *Bartonella* and *Rickettsia* in arthropods from the Lao PDR and from Borneo, Malaysia. Comp Immunol Microbiol Infect Dis. 2012;35:51–7. PubMed http://dx.doi.org/10.1016/j.cimid.2011.10.003

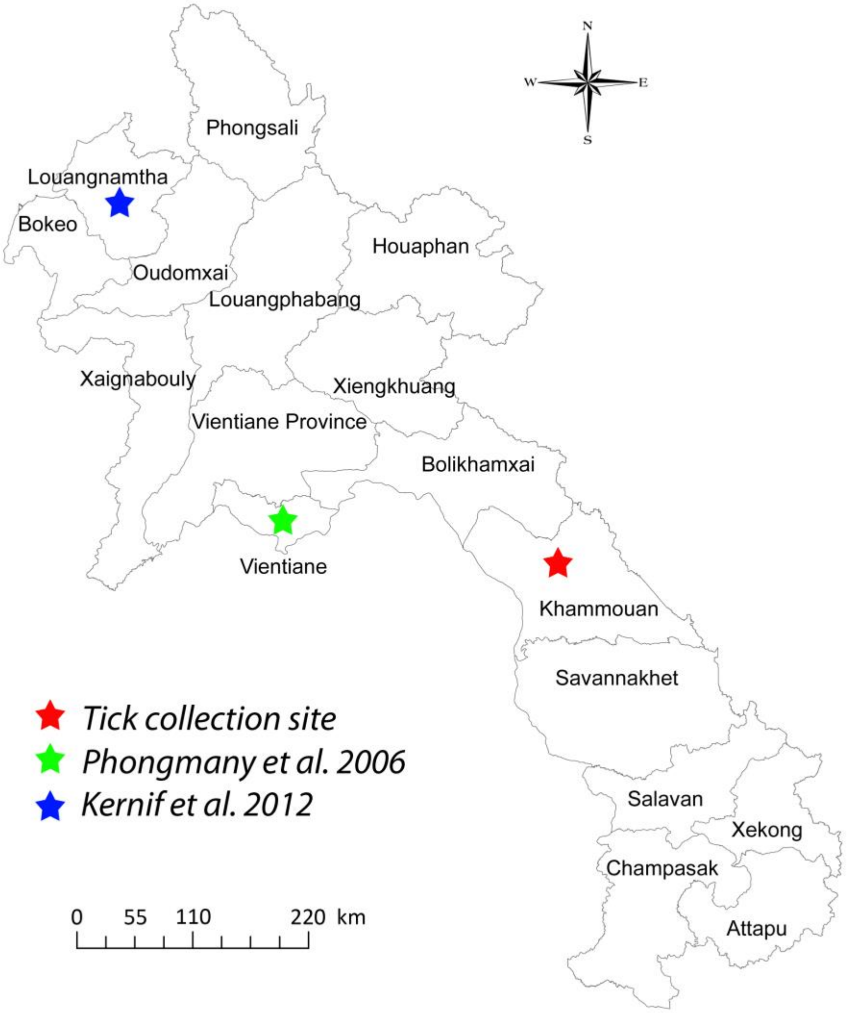

Technical Appendix Figure 1. Khammouan Province (red star), Laos, where ticks were collected in this study (see Technical Appendix Figure 2). Locations of previous studies investigating *Rickettsia* spp. in Laos are shown by the green star (*8*) and blue star (*9*).

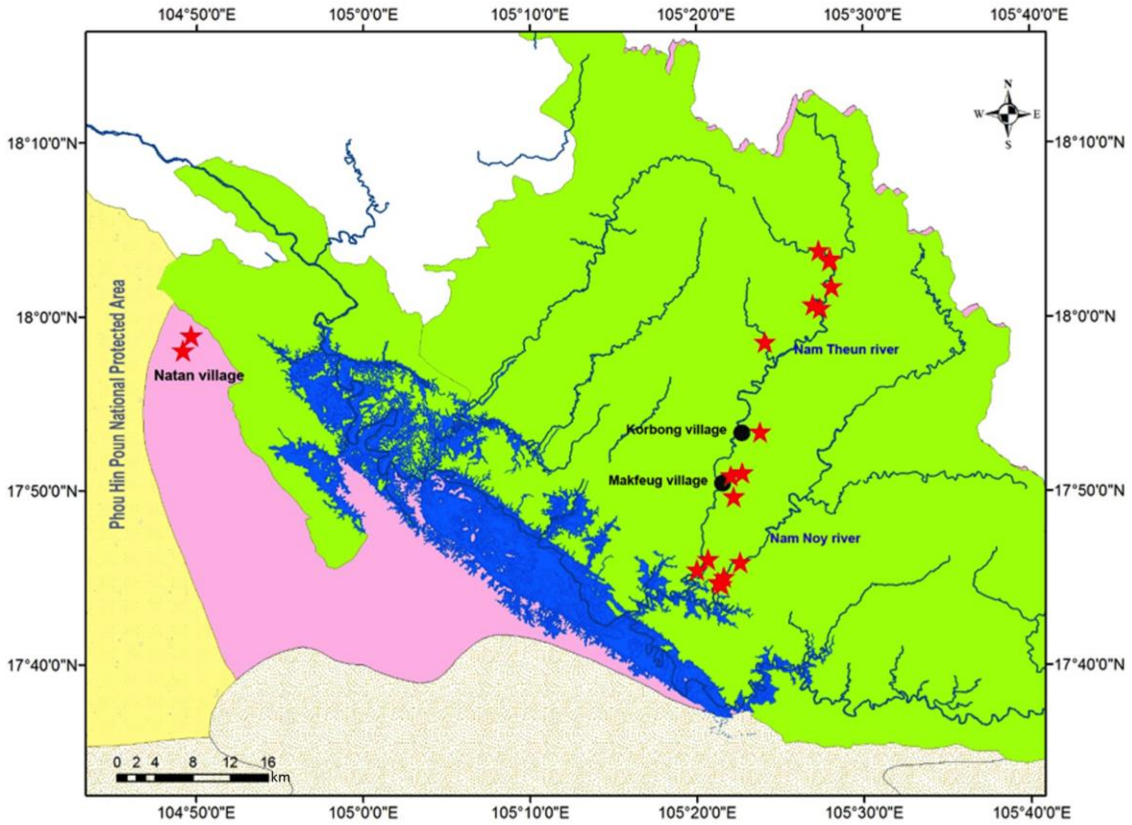

Technical Appendix Figure 2. Location of tick collections sites (red stars) in Khammouan Province, Laos.

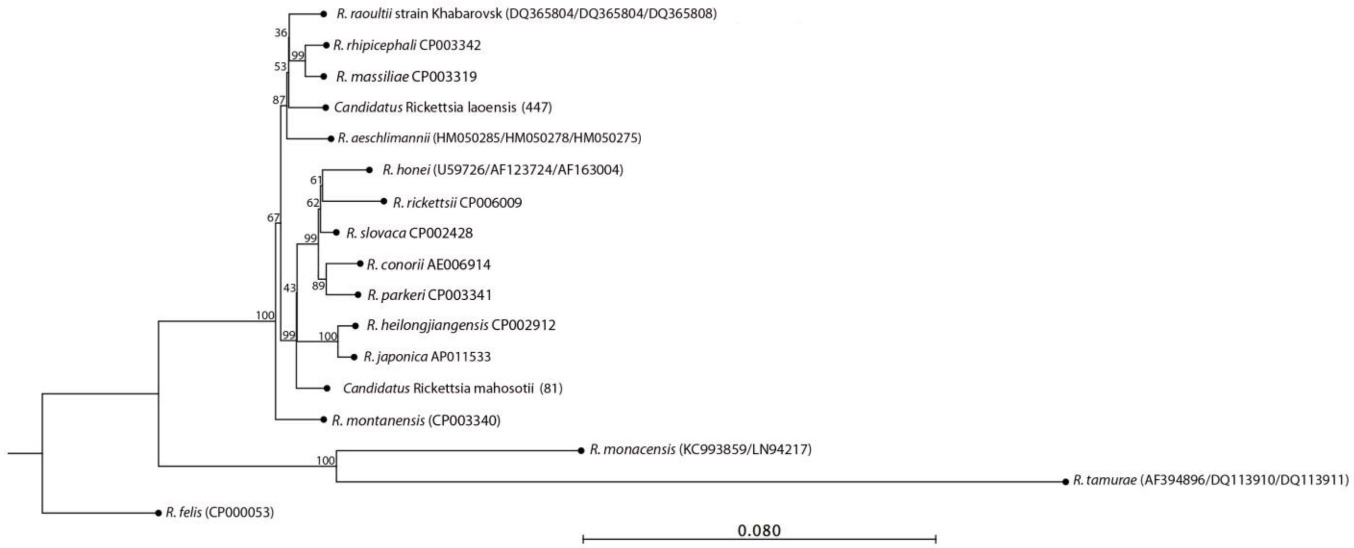

Technical Appendix Figure 3. Phylogenetic analysis of *gltA, sca4*, and *ompB* genes of candidate novel *Rickettsia* spp., Kammouan Province, Laos. The tree was constructed by using concatenated partial nucleotide sequences (2,845–2,920 bp) of *gltA*, *sca4*, and *ompB* genes; the Kimura-80 model; and the neighbor-joining method. Analyses were supported by using bootstrap analysis with 1,000 replications. Numbers along branches are bootstrap values. Sample numbers identifying each tick pool from this study are shown in parentheses after the sequence name. Scale bar indicates nucleotide substitutions per site.

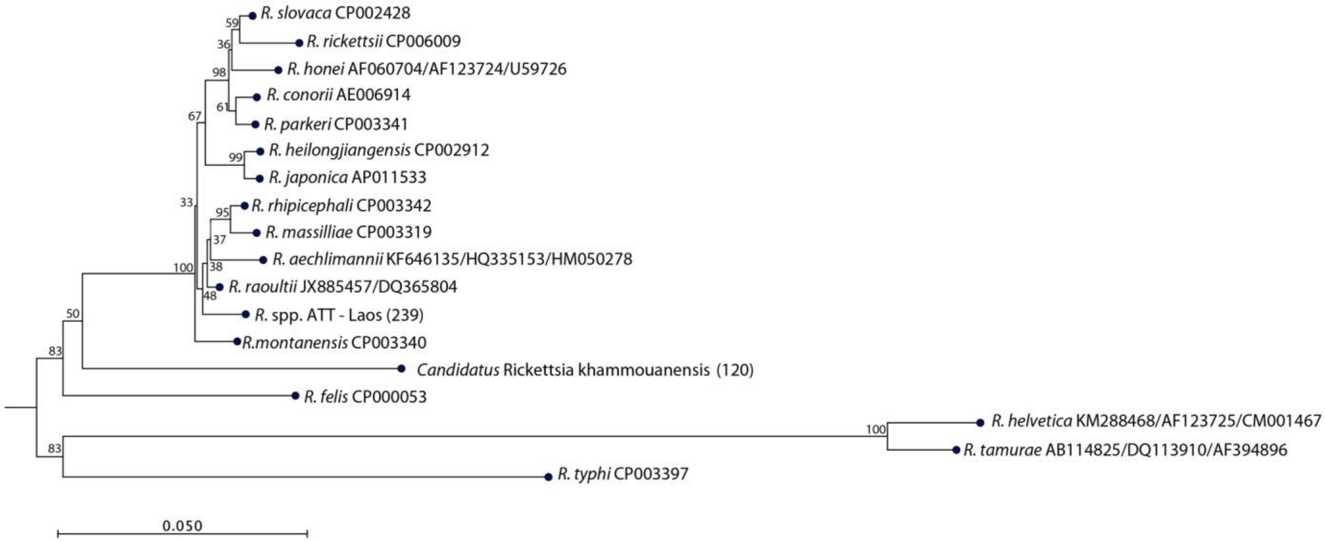

Technical Appendix Figure 4. Phylogenetic analysis of *gltA,* 17 kDa, and *ompB* genes of *Rickettsia* spp., Kammouan Province, Laos. The tree was constructed by using partial nucleotide sequences (1,114–1,117 bp) of concatenated sequences of *gltA,* 17-kDa, and *ompB* genes; the Kimura-80 model; and the neighbor-joining method. Analyses were supported by using bootstrap analysis with 1,000 replications. Numbers along branches are bootstrap values. Sample numbers identifying each tick pool from this study are shown in parentheses after the sequence name. Scale bar indicates nucleotide substitutions per site.
